# Supplementary material for: Prevalence and predictors of kidney dysfunction among people living with HIV in Tanzania
Source: PLOS Glob Public Health. 2025 Aug 1;5(8):e0005011. doi: 10.1371/journal.pgph.0005011 (PMC12316212; doi:10.1371/journal.pgph.0005011)
Supplement: Attachment — Submitted filename: PLOS public health.pdf [file pgph.0005011.s002.pdf]

PLOS public health

Response to Editorial Office – Manuscript ID: PGPH-D-25-00958R1

Thank you for your message and for identifying the concern regarding potentially identifying information in the supporting information file (“Temeke_2025.xlsx”).

In response to your request, we have reviewed the dataset and removed all potentially identifying variables. We have now uploaded a fully anonymized version of the dataset that complies with PLOS Global Public Health’s data sharing policy.

We appreciate your guidance in ensuring ethical standards and participant privacy, and we confirm that this de-identified dataset has been approved for sharing in accordance with the ethics approval obtained for this study.

Thank you for your continued consideration of our manuscript.

Please let us know if any further action is required.

Sincerely,
Dr. Daniel Msilanga
Corresponding Author
