# Supplementary material for: Prevalence and predictors of kidney dysfunction among people living with HIV in Tanzania
Source: PLOS Glob Public Health. 2025 Aug 1;5(8):e0005011. doi: 10.1371/journal.pgph.0005011 (PMC12316212; doi:10.1371/journal.pgph.0005011)
Supplement: Attachment — Submitted filename: Response to comments_July.docx [file pgph.0005011.s003.docx]

**Response to Reviewers Comments**

| **`Reviewer 1** | |
| --- | --- |
| Lines 71-72: Kindly reference the statement, “This study is a sub-analysis of data collected from a larger project titled “Point-of-Care Creatinine Testing for Early Detection of Renal Dysfunction in Tanzanian HIV Patients. | Thank you for the comment. Reference has been added to the sentence |
| Lines 144-145: Following the statement, “These variations likely reflect differences in patient characteristics, healthcare levels, and definitions of kidney dysfunction (15)”, kindly provide possible reasons why this study found the prevalence of kidney dysfunction of 15.6% among PLHIV, and others that found higher prevalences of 25% and 32.8% in similar urban hospital-based settings. | Our study results are comparable to the findings by Mapesi et al. and Mwanjala et al. The other studies by Msango used a higher  cut-off of estimated glomerular filtration rate of less than 90mls/min (As the definition of kidney dysfunction) compared to 60mls/min used in our study. This has been added to the manuscrit |
| **Reviewer 3** | |
| **Comments** | **Response** |
| Line 98 and Table 2: Unit of eGFR should be appropriately captured as milliliters per minute per 1.73 square meters (mL/min/1.73m²) throughout the manuscript. | Thank you for pointing this out. This has been corrected to reflect a correct format |
| Line 105, 106 and Table 3: Univariate and multivariate is used interchangeably with univariable and multivariable throughout the manuscript. Kindly stick to the use of univariable and multivariable | Thank you for pointing this out. This has been corrected. |
| Table 2: Kindly stick to the use of the abbreviation “eGFR” throughout the manuscript. | This has been corrected. |
| Table 3: “No - Ref” under Alcohol use and Comorbidities should be removed. | This has been corrected. |
| References: Ref 3 is incomplete | This has been corrected. |
